# Myrmecophilous Aphid Species (Hemiptera, Aphididae) Feeding on Mycoheterotrophic *Monotropa hypophegea* (Ericales, Ericaceae)

**DOI:** 10.3390/insects16010019

**Published:** 2024-12-28

**Authors:** Bartosz Bielecki, Patryk Długosz, Miłosz Morawski, Łukasz Depa

**Affiliations:** Institute of Biology, Biotechnology and Environmental Protection, Faculty of Natural Sciences, University of Silesia in Katowice, Bankowa 9, 40-007 Katowice, Poland

**Keywords:** *Aphis*, parasitism, phytophagy, mutualism, trophobiosis, ecology, pollination

## Abstract

The paper presents the first report and morphological description of aphids feeding on the mycoheterotrophic plant *Monotropa hypophegea* Wallr. of the family Ericaceae. This is the first known case of aphids feeding on a mycoheterotrophic plant, additionally involved in mutualistic relation with ants *Lasius niger* (Hymenoptera, Formicinae). This observation took place on an overgrown, postmining spoil tip in southern Poland (Europe). Barcode COI sequencing and morphological examination indicated that the collected specimens of aphids belong to *A. fabae* s. lat., a group of closely related species. The discussion focuses on taxonomic issues concerning the species identity of the collected aphids and the ecological interactions between insects, plants, and fungi involved in the observed association. The presence of ants attending aphids indicates the possibility of ants serving as pollinators of *M. hypophegea*.

## Simple Summary

Aphids are pests worldwide, but also an important part of ecosystems in temperate zones, as insects feeding on chlorophyllous plants. There are, however, plants that have lost their ability to photosynthesize and obtain nutrients from other plants via their symbiotic fungi. There are few accounts of aphids feeding on such plants, and here we present the case of an aphid species not only feeding on achlorophyllous, parasitic plants but also involved in mutualistic relation with ants. We discuss the results in respect to aphid taxonomy, parasitism and connections between various species–plants, fungi and insects involved in this interaction.

## 1. Introduction

Aphids (Insecta, Hemiptera, Aphididae) are sap-sucking insect parasites of plants and are among the most significant plant pests worldwide. They cause direct damage to plants by exploiting assimilates through the acquiring of a phloem sap, but they are also vectors for many viral diseases of agricultural and wild plant species [1,2]. Being nutritionally dependent on the products of photosynthesis, they are predominately connected to plants having functional chlorophyll, constituting the majority within this kingdom of organisms, and their primordial adaptative trait, photosynthesis. Parasitic plant species constitute about 1% of all angiosperm species [3] and aphids are rarely found on them, usually on species which still have chlorophyll (hemiparasites), e.g., *Tuberaphis coreana* Takahashi, 1933 on mistletoe *Viscum album* [4], or on heterotrophic hosts that parasite directly on other plants, e.g., *Smynthurodes betae* Westwood, 1849 on broomrape *Phelipanche ramosa*, which is a pest to agriculturally important Solanaceae species [5]. The rarity of aphids feeding on holo-parasitic plants may result from difficulties in collecting, e.g., the short periods of occurrence of such plants, lasting only a few months, but also from difficulties with the infestation of plants by aphids for a relatively short time, e.g., a few weeks to a few months of flowering, or inappropriate time of occurrence of the plant, e.g., early spring. In this respect, special attention must be given to mycoheterotrophic plants, which may be considered parasites of plants via mycorrhizal fungi [6]. Most such plants belong to the family Ericaceae, particularly the subfamily Monotropoideae, known as monotropes [7]. These are herbaceous perennials with underground stems and roots living in ectomycorrhizal association with fungi, which are in mutualistic mycorrhizal association with plants. They lack chlorophyll, so the mono-tropes are regarded as indirect epi-parasites of other plants, probably deriving carbohydrates from other plants via fungi [8]. The mode of deriving nutrients by mycoheterotrophic plants may also be a reason for no previous observation of aphids on these plants, possibly as nutritionally insufficient hosts for fast reproducing aphids, requiring a significant amount of sap.

Here, we present data on ant-attended aphids on flowering shoots of *Monotropa hypophegea* Wallr. 1822, a common but relatively rarely collected mycoheterotrophic plant species. The aphids were similar to *Aphis vaccinii* (Börner, 1940), a species feeding on non-parasitic Ericaceae, and *A. podagrariae* Schrank, 1801, feeding on *Aegopodium podagraria* and rarely on other Apiaceae, but differed slightly in their morphology and barcode sequence. We discuss observed differences of collected specimens in a taxonomical context, as well as regarding implications for the ecological interrelationship between ants, aphids and plants involved in interspecific association.

## 2. Material and Methods

The study and the first collection took place on 7 July 2022 in a pine forest (*Pinus sylvestris*) overgrowing the XIX century spoil tip after dolomite mining in Tarnowskie Góry (Poland): 50°24′54.8″ N 18°51′19.7″ E. The next collection from the same site took place on 18 July 2023.

Additionally, during consecutive studies, there was another collection of aphids from *M. hypophegea* at Miasteczko Śląskie (50°30′7.31″ N 18°54′29.60″ E) in a similar pine forest on 18 July 2023.

The first author collected stems of *M. hypophegea* Wallr. (syn. *Monotropa hypopitys* L.–widely accepted name, but not following observed differences between the two forms, *M. hypopitys* and *M. hypophegea*, both observed during the studies, but with ants only on *M. hypophegea*; *Hypopitys hypophegea* (Wallr.) G. Don) during his botanical studies, noticing the ants attending it, and stored this at −15 °C for a few months. After re-examining the collected plant, he discovered specimens of aphids, which were preserved in 70% and 99% ethanol for the senior author to identify. The following collections took place in a similar manner.

Aphids collected in 70% ethanol were mounted following the protocol by Kanturski and Wieczorek [9] and determined using a key by Heie [10]. The morphological description follows the mode widely accepted by aphidologists (e.g., Heie) [10]. Studied specimens were imaged using a Nikon Eclipse E-600 biological microscope with a Nikon DS-Fi2 digital camera and NIS Elements software (version 4.2, Nikon, Japan). Microscopic slides are deposited in the entomological collection of the University of Silesia in Katowice (DZUS).

The following additional material was studied for morphological comparison of the collected specimens:

*Aphis* (*Aphis*) *podagrariae* Schrank, 1801:

26 June 1965. Olsztyn, Kortowo; *Aegopodium podagraria*, leg., det. H. Szelegiewicz, R690, 882; 1 adult apterous viviparous female;

1 June 2004. Skała: Ojcowski Park Narodowy, Młynnik; *Aegopodium podagraria*, leg., det. B. Osiadacz. SA02-31-83-009 DZUS; 4 adult apterous viviparous females;

11 June 2005. Piekary Śląskie: Dąbrowa 1. *Aegopodium podagraria*; *Lasius niger*, *Myrmica rubra*, leg., det. Ł. Depa, SA02-31-83-015 DZUS; 4 adult apterous viviparous females;

*Aphis vaccinii* (Börner, 1940):

23 July 1965. Celestynów, distr. Otwock, Polonia; *Vaccinium uliginosum*, leg., det. H. Szelegiewicz, R884, 2690; 5 adult apterous viviparous females;

7 July 1960. wrzosowisko Bielawskie Błota; *Andromeda polifolia*, leg., det. H. Szelegiewicz, R863, 1302; 3 adult apterous viviparous females;

9 July 1964. Olsztyn, Kortowo; *Vaccinium uliginosum*, leg., det. H. Szelegiewicz, R864, 135/94; 1 adult apterous viviparous female.

**Figure 1:**
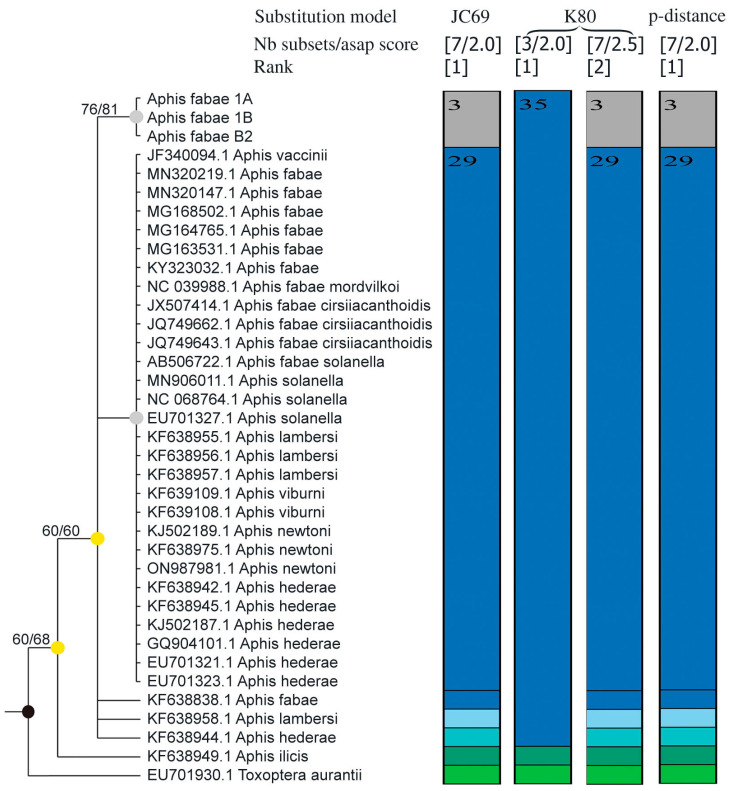
Phylogenetic tree based on COI marker including collected specimens (voucher numbers 1A, 1B and B2) and sequences of related species from Genbank (accession numbers presented) and ASAP partitioning (bars of different color indicate different sequences, possibly of different species) depending on applied substitution model (numbers on tree nodes represent bootstrap values for K2P model and p-distance, respectively).

Specimens of newly collected aphids (3 individuals, single from each collection) preserved in 99% ethanol served to obtain the COI barcode sequence (using standard barcode LCO1490 and HCO2198 markers). DNA was extracted using a DNeasy Blood and Tissue Kit by Qiagen (Venlo, Netherlands), and a standard barcode sequence of mitochondrial cytochrome c oxidase subunit I (COI) was amplified using the primers LCO1490 and HCO2198. Kimura 2-parameter model in MEGA11 was used to determine the molecular distance between the sequence of newly collected aphid specimens and other species belonging to the *Aphis fabae* group (Genbank accession numbers presented in Figure 1). To resolve the relationship of collected specimens, the phylogenetic trees were obtained using MEGA11, applying the Kimura 2-parameter and p-distance models for Neighbour-Joining.

The DNA-based species delimitation method ASAP (Assemble Species by Automatic Partitioning [11], already applied for aphid species separation [12], was used to confirm the taxonomic status of the discovered specimens. It was performed using the ASAP web server (https://bioinfo.mnhn.fr/abi/public/asap/, accessed on 12 December 2023) with the alignment of sequences used for Neighbour-Joining analysis as an input file.

## 3. Results

**Figure 2:**
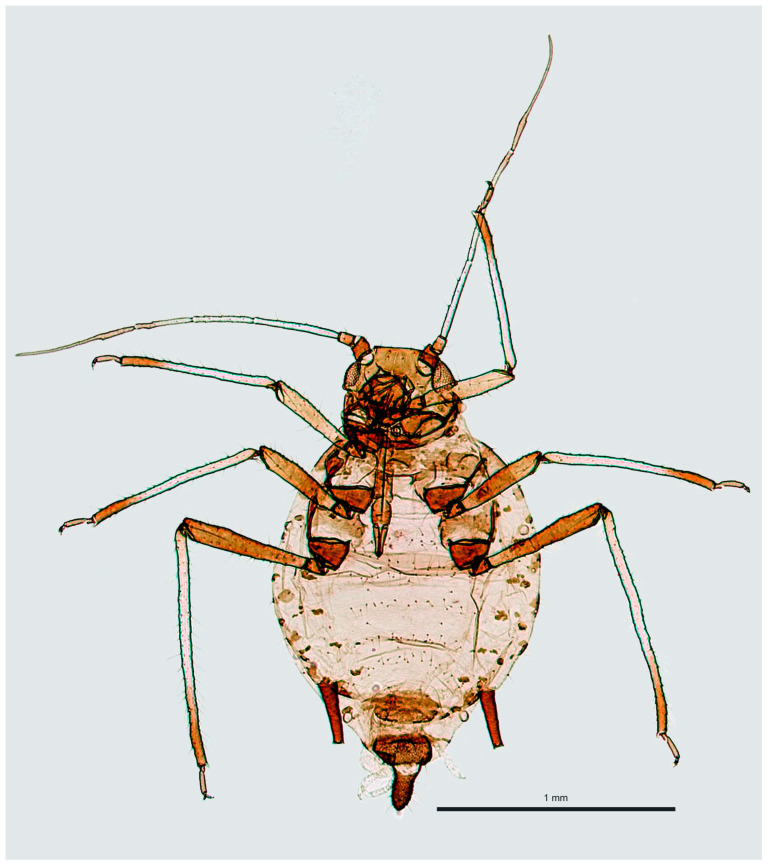
Specimen of the *Aphis fabae* collected from *Monotropa hypophegea* on 18 July 2023 in Miasteczko Śląskie.

The molecular analysis allowed us to obtain a 545 bp long COI sequence of collected aphids. It allowed the placing of the specimen within the *Aphis fabae* group of species of the subfamily Aphidinae, tribe Aphidina (Figure 1). The genetic distance between COI sequences of the studied specimens and other representatives of the *A. fabae* group was comparable to distances between other species and subspecies within this group (Table 1), although very low. Nevertheless, specimens collected from *M. hypophegea* clustered within the single clade (Figure 1 and Figure 2).

The ASAP analysis, via various methods (Figure 1), distinguished collected specimens as separate species. Among the three models applied, the two with the lowest score (JC69 and p-distance) separated sequences of collected specimens as a separate species with the lowest ASAP scores while, with the application of the K80 model, the results with the lowest ASAP score distinguished only three species, including collected specimens, into *A. fabae* group, but the second-lowest score separated them into a single cluster.

Morphological analysis of the mounted specimens (seven adult apterous viviparous females) showed their close resemblance to *Aphis vacinii*, an oligophagous species feeding on autotrophic Ericaceae, but also to *Aphis podagrariae*, a monophagous species feeding on *Ae. podagraria* and rarely on other Apiaceae. Certain small but clear differences in morphology were noticed, which is in line with the results of molecular studies, while maintaining the generally high morphological similarity of species to the *A. fabae* group.

### 3.1. Morphological Characteristics

Material studied: four apterous viviparous females (Figure 2) from Miasteczko Śląskie;

fhree apterous viviparous females from Tarnowskie Góry.

#### Description

**Figure 3:**
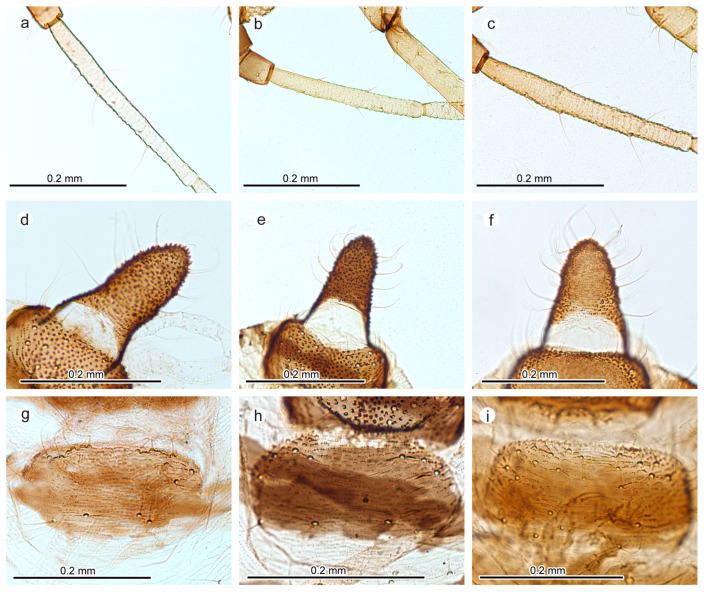
Morphological details of the studied specimens of *A. fabae* collected from *Monotropa hypophegea* (**a**,**d**,**g**), *A. vaccinii* (**b**,**e**,**h**) and *A. podagrariae* (**c**,**f**,**i**); antennal segment III (**a**–**c**), cauda (**d**–**f**), genital plate and its setae (**g**–**i**).

Body in life, dark brownish to black; in mounted specimens, cuticle weakly pigmented, with darker (but not black) head, femora, tips of antennae, tibiae, and slightly darker siphunculi and cauda; sclerotization weak, with no traces of spinal or pleural sclerites, only small marginal sclerites, post-siphuncular sclerites and a small crossbar on abdominal tergite VII; intersegmental muscle sclerites also visible; cuticle covered with delicate rows of small spinules, sometimes arranged hexagonally, visible only in distal part of the abdomen; body length 1.68–1.91 mm; antenna six-segmented, without secondary rhinaria (Figure 3a), 1.19–1.47 mm long, 0.66–0.84 of body length, VIb/VIa 2.42–2.93; longest seta of antennal segment III 1.73–2.80 of IIIbd; rostrum 0.56–0.71 mm long, 0.29–0.39 of body length, ultimate rostral segment 0.13–0.16 mm long with two accessory setae, 1.20–1.26 of the second segment of hind tarsus. The longest posterior seta of the middle trochanter is 1.18–1.73 of the trochanter–femoral suture, and the longest anterior seta of the middle femur is 1.25–1.60 of this suture. Marginal seta of abdominal segment I 2.4–3.4 of the height of marginal tubercle of this segment, one to four marginal tubercles on abdominal segments II–VI. Abdomen membranous, with sclerotic cross-bars only on tergite VII and VIII; the last one with 2–4 setae 0.056–0.080 mm long; diameter of the marginal tubercle of abdominal segment VII 1.29–2.20 of IIIbd. Siphunculi imbricate, tapering, as dark as cauda, slightly darker than the head, femora, and tips of tibiae; 0.20–0.25 mm long, 0.11–0.13 of body length, 0.93–1.16 of cauda length. Cauda is rather tongue-shaped, mostly with a slight constriction in the middle (Figure 3d), 0.18–0.24 mm long, 0.068–0.092 mm wide in the middle, with 16–19 setae; its width in the middle is 0.35–0.45 of its length. Genital plate oval, as dark as cauda, 0.11–0.14 mm long and 0.26–0.27 mm wide, with 3–5 anterior and 12–14 posterior setae, along its distal margin (Figure 3g).

**Figure 4:**
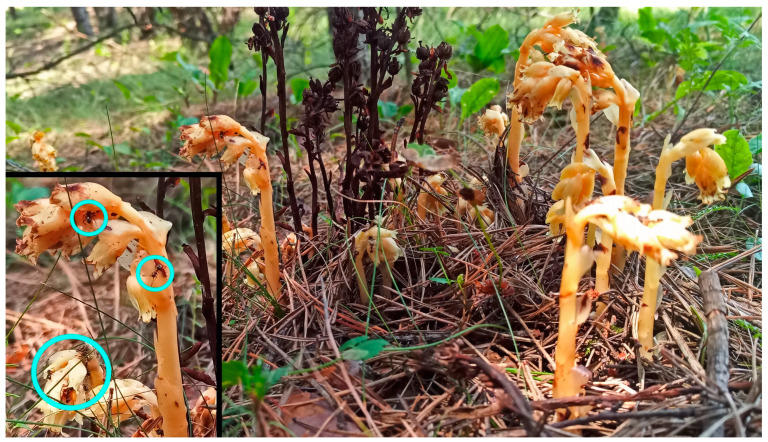
Stems and inflorescences of *M. hypophegea* in Tarnowskie Góry (circles mark the presence of ants attending aphids).

Biology: Aphids were found feeding on the stems and pedicels of flowers and inside the flowers of *M. hypophegea*. In Tarnowskie Góry, they were located on three of sixteen inflorescences of host plants, visited by ants of the species *Lasius niger*, in 2022 (Figure 4), and on two of three inflorescences in 2023. In Miasteczko Śląskie in 2023, the aphids were found on four of forty-three inflorescences.

### 3.2. Taxonomic Comments

In general appearance and in most morphological traits, the collected specimens may be placed well within the range of variability of the *A. fabae* species group, as also proven by molecular studies. This is a very complex and taxonomically difficult group of species, with a high level of morphological similarity and intraspecific variability, possible existence of many host races or lineages and possible hybridization. The general feature distinguishing newly observed aphids from *A. fabae* s. lat. is the constant presence of at least one marginal tubercle on abdominal tergites II–VI, a tongue-shaped but relatively elongated cauda with weak constriction in the middle (Figure 3d vs. Figure 3e,f), and very weak sclerotization, with pale cross-bar only on abdominal tergite VII, small, pale post-siphuncular sclerites and very small, pale marginal sclerites. This sclerotization pattern concerned all studied specimens of *A. fabae* from *M. hypophegea*, regardless of collection site and year while, in other representatives of the *A. fabae* group, the sclerotization pattern is variable even within the single colony.

Among the examined species, morphologically, *A. fabae* from *M. hypophegea* most resembles *A. vaccinii* and *A. podagrariae*. However, a series of distinguishing traits may be defined, as presented in Table 2. The two main morphological traits allowing distinction of the three species are (1) the ratio of the diameter of cauda in the middle to the length of longest seta on abdominal tergite VIII, which is lowest in *A. fabae* from *M. hypophegea* due to relatively elongated and slightly constricted cauda in comparison to tongue-shaped cauda in *A. vaccinii* and *A. podagrariae* (and generally *A. fabae*) (Figure 3e,f); (2) the number of setae on the distal edge of the genital plate, most numerous in *A. podagrariae* (Figure 3i) and least numerous in *A. vacinii* (Figure 3h), with *A. fabae* from *Monotropa* placed in between (Table 2, Figure 3g). Another feature distinguishing it from *A. vaccinii* and *A. podagrariae* is the ratio of rostrum length to width of the genital plate, which in the latter two is lower than 2.00 while in *A. fabae* from *Monotropa* is higher than 2.08, mainly due to the relatively longer rostrum in the specimens of newly recorded colonies. Other distinctive features include the length of setae on antennal segment III (Figure 3b,c) and their relation to the diameter of cauda in the middle, especially distinguishing it from *A. vaccinii* (Table 2).

## 4. Discussion

Despite the closest resemblance to *A. podagrariae*, the specimens infesting *M. hypophegea* cannot be regarded as this species (apart from morphological and molecular differences) due to its strict trophic association with *Ae. podagraria* and rarely with other Apiaceae, so far unknown to infest any Ericaceae. All species within the *A. fabae* group are very similar morphologically and have the potential to interbreed and develop host plant races, which poses difficulties in resolving their taxonomic status [13]. They are also facultatively myrmecophilous species, often not attended by ants. Although the existence of the *A. fabae* group is confirmed, the differentiation of species within the group may rely more on the basis of ecological traits (e.g., host plant affiliation) than solely on morphological or molecular data [14,15]. In this case, we obtained a low but stable molecular distance of *A. fabae* from *M. hypophegea* compared to other representatives of *A. fabae* group (Table 1), much lower than typical in the genus *Aphis* (e.g., Cocuzza and Cavalieri [16]), but not unprecedented within the species groups, e.g., *A. sedi* vs. *A. gossypii* 0.08–0.70% [17] or *A. eupatorii* and *A. teucrii* vs. *A. frangulae*, 0.3% and 0.4%, respectively [16]. Molecular data, as presented in Figure 1, clearly show uncertainties regarding species distinction, with mitochondrial barcode COI within the *A. fabae* complex, sustained by difficulties in proper morphological determination of samples used for molecular studies. Therefore, despite small but stable morphological and molecular differences and the overall similarity of collected specimens to *A. fabae*, distinguishing the collected specimens at most as a lineage within *A. fabae* is the most reasonable solution until the complex taxonomic revision of the *A. fabae* group is performed. *A. fabae* belongs to the most widely distributed and most polyphagous aphid species worldwide, aprone to phenotypic plasticity [18], which also may be a case here. Its presence on mycoheterotrophic *M. hypophegea* may not be surprising, especially when its primary host plant, *Euonymus* sp., is a widely distributed native plant species and early infestation, soon after the emergence of spring buds. of *M. hypophegea* is very plausible.

Following the existing database on plant–aphid relationships [4], there are no accounts of aphids feeding on *M. hypophegea* or other Monotropeae so, even for polyphagous *A. fabae*, this is the first account of its occurrence on this host plant. The closest host plant relatives are Pyroleae, on which a single aphid species, *Aulacorthum rufum*, was found, and also in Poland and north-western Russia [19]. However, this is not a close relative of *A. fabae* as it belongs to a separate tribe, Macrosiphini, while the collected species belongs to the tribe Aphidini. The lack of records of aphids on mono-tropes may result from a few factors: short time of occurrence of available host plants (in 2023, the first stems of *M. hypophegea* were emerging from the ground on 30 May), insufficient for proper collection of aphids by aphidologists, or insufficient for infestation of plants by dispersing aphids. The reason may also be the unsatisfactory nutritional value of mycoheterotrophic plants for aphids, which require a vast quantity of phloem sap for proper growth and reproduction (approximating half of the body weight per hour [20]). The collected aphids lived in colonies consisting of several specimens per inflorescence, adults and larvae, which proves their ability to gain enough nutritional elements to reproduce parthenogenically. In addition, no winged morphs serving for dispersal were found, which suggests that these colonies started at least a few weeks earlier if funded by dispersing alate females.

The presence of ants attending recorded aphid colonies is also an important feature, as many aphids are connected to ants by trophobiotic mutualism [21], where aphids gain shelter and protection from ants in turn for excreted honeydew, providing ants with carbohydrates and other nutrients. Ant-attended aphids feeding on roots and tree trunks may also survive winter hidden in ant chambers under the bark [22]. While the experiments show that light deprivation of plants infested by aphids increased aphid mortality [23], theoretically this may not concern mycoheterotrophic host plants, which may provide aphids with nutrients via underground roots throughout the season, obtaining nutrients from fungi. This would be a valuable feature of such plants in terms of host plant specialization of aphids during their evolution as a more stable resource for exploitation. In many cases, ants may transfer aphids to more profitable locations, e.g., from roots to leaves and vice versa [24]. In this case, despite *A. fabae* being a generally host-alternating (with common *Euonymus* as a leading primary host plant) and facultative myrmecophilous species, aphids may persist throughout the year, either on inflorescence or on the roots of *M. hypophegea* with the assistance of ants. Although feeding on roots is not reported in the case of *A. fabae*, it occurs in other species of the genus *Aphis*, e.g., *A. sambuci* [4], and then it is usually assisted by attending ant workers. The existence of permanent parthenogenetic generations in *A. fabae* on the secondary host throughout the year is known [25], so there is a certain probability that this may also concern aphids on *Monotropa*, especially if they are constantly ant-attended. While this relationship may seem inviable for *M. hypophegea*, it may not be so if ants in this way provide the plant with some beneficial service, e.g., by pollination, due to the fact that many aphids were located inside the flowers. So far, little is known about the pollination of monotropoid plants, indicating bumblebees and other hymenopterans as potential pollinators [26,27]. In this case, ants looking for aphids and visiting many flowers and neighboring inflorescences might increase the chances for self-pollination of *M. hypophegea*. If so, infestation by myrmecophilous aphids may contribute to the reproductive success of the monotropoid plant, as it may not be connected with additional loss of nutrients provided by fungus. Such a benefit for plants hosting ant-attended aphids is possible. This has been noted in several cases, where e.g., ants visiting aphid colonies protect the infested plant from herbivory by other arthropods, which serve ants as a source of proteins [2,28]. Although ants are generally regarded as rare pollinators, this may not be a strict rule because, in some cases, they may contribute to certain plants’ pollination [29] and autogamy [30].

**Figure 5:**
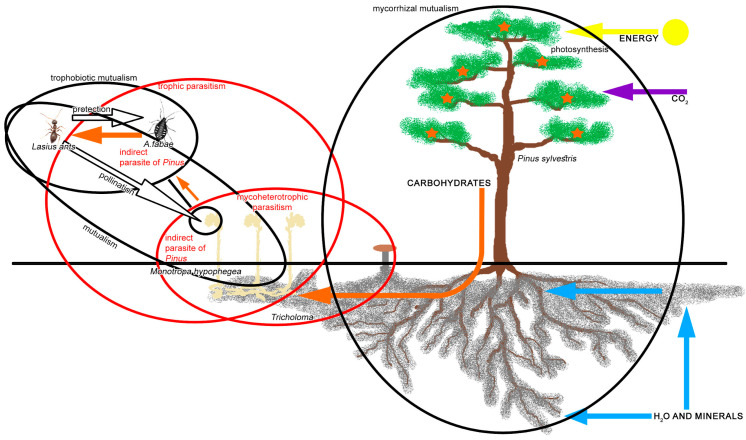
A scheme of the ecological interrelations between ants attending aphids feeding on *M. hypophegea* and the relationship with photo-synthesising plants via mycorrhizal fungus (horizontal arrows indicate the inflow of inorganic substances and energy, orange arrows indicate flow of organic substances/nutrients, black arrows indicate ecological services, dark circles indicate symbiotic relations, red circles indicate parasitic relations).

The observed mutualistic relationship of ants *L. niger* and aphids *A. fabae* feeding on mycoheterotrophic *M. hypophegea* should be analyzed within a broader ecological view of arthropod-plant interactions. While aphids are generally considered parasites of plants, in this case it should not be as easy to consider *A. fabae* as a direct parasite of *M. hypophegea*, because the latter does not provide nutrients originating from its assimilation apparatus [31]. While the biochemical composition of the phloem of *M. hypophegea* may be different than its original plant source (*Aphis* spp. do not feed on *Pinus* spp. and other conifers), the chlorophyllous plant is still an original source of organic substances as resources utilized by mycoheterotrophic plants. If *M. hypophegea* obtains nutrients from chlorophyllous plants via their mycorrhizal fungi, then the aphids which feed on *M. hypophegea* might be regarded as direct parasites of *M. hypophegea*, but also indirect parasites of the original chlorophyllous host plant [32]. Following this scheme (Figure 5), if the ants are involved in a mutualistic relationship with aphids as a dominant resource, by acquiring carbohydrates from these aphids, they should be taken indirectly from the original source, which is the chlorophyllous mycorrhizal plant. Such ants may be regarded then as the facultative parasites of the chlorophyllous plant. In this context, we have two mutualistic relationships: mycorrhizal fungus–tree and trophobiotic ant–aphid, connected by parasitic relationships of *M. hypophegea* and feeding aphids. However, for *M. hypophegea*, this aphid-mediated relation with ants may be beneficial and symbiotic if ants serve as pollinators or protect it from herbivory [33]. Although, in the latter case, a low nutritional value of such plants could protect them from herbivory, e.g., from folivores [34], it seems that, at least for polyphagous aphids, *M. hypophegea* provides sufficient nutrition to sustain a colony under ant-attendance.

The relationship of aphids with parasitic and especially mycoheterotrophic plants has not been satisfactorily studied. This deficiency of detailed observations makes it impossible to determine how frequent such a relation is and whether it is an occasional, random co-occurrence, as seems to be in the case with reported *A. fabae* on *M. hypophegea*, or if some more strict interdependence occurs. The extreme rarity of such reports may result from observational problems, but this does not mean such cases are rare; rather, they seem to be under-recorded. In addition, the ecosystem services of ant-aphid mutualism on mycoheterotrophic plants remains puzzling due to the possibility of self-pollination by ants. Finally, whether any kind of host plant specialization of aphids exists regarding mycoheterotrophic, achlorophyllous species remains an open issue.

## Figures and Tables

**Table 1:** Genetic distances between sequences of *A. fabae* from *M. hypophegea* and other species of *A. fabae* group calculated using the K2p model (expressed as a percentage).

	Mean	Minimum	Maximum
*A. fabae* from *Monotropa*	0.00	0.00	0.00
*A. f.* from *Monotropa* vs. *A. vaccinii*	0.23	0.23	0.23
*A. f.* from *Monotropa* vs. *Aphis fabae*	0.24	0.23	0.45
*A. f.* from *Monotropa* vs. *A. fabae* group	0.27	0.23	0.68
*A. fabae*	0.03	0.00	0.23
*A. fabae* vs. *A. fabae* group	0.07	0.00	0.68
*A. fabae* group without *A. fabae*	0.08	0.00	0.68
*Toxoptera aurantii* vs. all other	6.76	6.72	7.00

**Table 2:** Set of morphological traits distinguishing *A. fabae* from *M. hypophegea* from *A. vaccinii* and *A. podagrariae* (HT2,, 2nd segment of hind tarsus, ant III, antennal segment III; / represents the proportion of traits; values distinctive for particular species are underlined).

	*A. fabae* from *Monotropa*	*A. vaccinii*	*A. podagrariae*
diameter of cauda in the middle/length of longest seta on abdominal tergite VIII	0.85–1.57	2.00–3.00	1.47–2.06
number of distal setae on genital plate	12–14	6–11	16–21
ultimate rostral segment/length of HT2	1.20–1.26	0.95–1.10	1.07–1.27
middle femur longest anterior seta length/III bd	1.06–1.52	1.63–2.50	1.18–2.12
length of longest setae on ant III/length of middle trochanter posterior seta	0.63–0.91	0.29–0.47	0.45–0.78
cauda mid diameter/length of longest seta on ant III	1.21–2.32	2.80–4.80	1.65–2.94
ultimate rostral segment/genital plate length	1.01–1.24	0.89–1.29	0.79–0.95
Cauda length/cauda mid diameter	0.35–0.45	0.36–0.60	0.48–0.72
length of rostrum/width of genital plate	2.08–2.76	1.59–2.00	1.57–2.01
middle femur longest anterior seta length/length of longest seta on abdominal tergite VIII	0.75–1.13	1.00–1.50	1.05–1.40

## Data Availability

Data are available from the corresponding author under request.
